# Association of Remdesivir Treatment With Survival and Length of Hospital Stay Among US Veterans Hospitalized With COVID-19

**DOI:** 10.1001/jamanetworkopen.2021.14741

**Published:** 2021-07-15

**Authors:** Michael E. Ohl, Donald R. Miller, Brian C. Lund, Takaaki Kobayashi, Kelly Richardson Miell, Brice F. Beck, Bruce Alexander, Kristina Crothers, Mary S. Vaughan Sarrazin

**Affiliations:** 1 Center for Access & Delivery Research and Evaluation, Iowa City Veterans Affairs (VA) Health Care System, Iowa City; 2 Department of Internal Medicine, Carver College of Medicine, University of Iowa, Iowa City; 3 Center for Healthcare Organization & Implementation Research, VA Bedford Health Care System, Bedford, Massachusetts; 4 Center for Population Health, Department of Biomedical & Nutritional Sciences, University of Massachusetts, Lowell; 5 VA Puget Sound Health Care System, Seattle, Washington; 6 Department of Internal Medicine, University of Washington, Seattle

## Abstract

**Importance:**

Randomized clinical trials have yielded conflicting results about the effects of remdesivir therapy on survival and length of hospital stay among people with COVID-19.

**Objective:**

To examine associations between remdesivir treatment and survival and length of hospital stay among people hospitalized with COVID-19 in routine care settings.

**Design, Setting, and Participants:**

This retrospective cohort study used data from the Veterans Health Administration (VHA) to identify adult patients in 123 VHA hospitals who had a first hospitalization with laboratory-confirmed COVID-19 from May 1 to October 8, 2020. Propensity score matching of patients initiating remdesivir treatment to control patients who had not initiated remdesivir treatment by the same hospital day was used to create the analytic cohort.

**Exposures:**

Remdesivir treatment.

**Main Outcomes and Measures:**

Time to death within 30 days of remdesivir treatment initiation (or corresponding hospital day for matched control individuals) and time to hospital discharge with time to death as a competing event. Associations between remdesivir treatment and these outcomes were assessed using Cox proportional hazards regression in the matched cohort.

**Results:**

The initial cohort included 5898 patients admitted to 123 hospitals, 2374 (40.3%) of whom received remdesivir treatment (2238 men [94.3%]; mean [SD] age, 67.8 [12.8] years) and 3524 (59.7%) of whom never received remdesivir treatment (3302 men [93.7%]; mean [SD] age, 67.0 [14.4] years). After propensity score matching, the analysis included 1172 remdesivir recipients and 1172 controls, for a final matched cohort of 2344 individuals. Remdesivir recipients and matched controls were similar with regard to age (mean [SD], 66.6 [14.2] years vs 67.5 [14.1] years), sex (1101 men [93.9%] vs 1101 men [93.9%]), dexamethasone use (559 [47.7%] vs 559 [47.7%]), admission to the intensive care unit (242 [20.7%] vs 234 [19.1%]), and mechanical ventilation use (69 [5.9%] vs 45 [3.8%]). Standardized differences were less than 10% for all measures. Remdesivir treatment was not associated with 30-day mortality (143 remdesivir recipients [12.2%] vs 124 controls [10.6%]; log rank *P* = .26; adjusted hazard ratio [HR], 1.06; 95% CI, 0.83-1.36). Results were similar for people receiving vs not receiving dexamethasone at remdesivir initiation (dexamethasone recipients: adjusted HR, 0.93; 95% CI, 0.64-1.35; nonrecipients: adjusted HR, 1.19; 95% CI, 0.84-1.69). Remdesivir recipients had a longer median time to hospital discharge compared with matched controls (6 days [interquartile range, 4-12 days] vs 3 days [interquartile range, 1-7 days]; *P* < .001).

**Conclusions and Relevance:**

In this cohort study of US veterans hospitalized with COVID-19, remdesivir treatment was not associated with improved survival but was associated with longer hospital stays. Routine use of remdesivir may be associated with increased use of hospital beds while not being associated with improvements in survival.

## Key Points

**Question:**

Is remdesivir treatment associated with improved survival or shortened hospitalizations among people with COVID-19 in routine care settings?

**Findings:**

In this cohort study of 2344 US veterans hospitalized with COVID-19, remdesivir therapy was not associated with improved 30-day survival but was associated with a significant increase in median time to hospital discharge.

**Meaning:**

The findings suggest that routine use of remdesivir may be associated with increased use of hospital beds but not with improvements in survival.

## Teaser

This cohort study assesses the association of remdesivir treatment with 30-day survival and length of hospital stay among US veterans hospitalized with COVID-19 between May and October 2020.

## Introduction

Remdesivir (GS-5734) is a prodrug of an inhibitor of the SARS-CoV-2 RNA-dependent RNA polymerase and was 1 of the first drugs studied for treatment of people with COVID-19.^1,2^ Randomized clinical trials have produced conflicting results about the efficacy of remdesivir.^3^ The Adaptive COVID-19 Treatment Trial (ACTT-1) found that remdesivir shortened the time to illness recovery from a median of 15 days to 10 days among patients hospitalized with COVID-19.^4^ Remdesivir treatment in ACTT-1 was not associated with a reduction in mortality at 28 days (11.4% vs 15.2%; hazard ratio [HR], 0.73; 95% CI, 0.52-1.03). The World Health Organization Solidarity Trial found that remdesivir treatment did not reduce the length of hospital stay or improve survival compared with the standard of care (rate ratio for death by 28 days, 0.95; 95% CI, 0.81-1.11).^5^ Other trials of remdesivir with varying designs have yielded equivocal results.^6,7^

Disparate trial results have led to conflicting recommendations regarding remdesivir use. The US Food and Drug Administration issued an emergency use authorization (EUA) of remdesivir treatment for patients hospitalized with COVID-19 in May 2020 and formally approved remdesivir in October 2020.^8,9^ The Infectious Diseases Society of America and the US National Institutes of Health treatment guidelines currently recommend remdesivir treatment for people hospitalized with severe COVID-19.^10,11^ These recommendations are partly based on the belief that if remdesivir use can shorten recovery time, it may allow more rapid discharge of patients from hospitals and open scarce beds to treat more patients during the pandemic. In contrast, the World Health Organization COVID-19 guidelines emphasize the lack of a survival benefit associated with remdesivir and recommend against the use of remdesivir for hospitalized patients.^12^

Observational studies can provide useful information about outcomes associated with remdesivir treatment in routine clinical practice. The Veterans Health Administration (VHA) is the largest integrated health care system in the US, with more than 6 million veterans in care in 2019.^13^ After the EUA and before US Food and Drug Administration approval of remdesivir, the VHA Pharmacy Benefits Management (PBM) created a centralized system to distribute remdesivir to VHA hospitals nationwide.^14^ As of October 1, 2020, VHA PBM had distributed remdesivir to treat more than 2500 patients with COVID-19, creating an opportunity to study outcomes of remdesivir treatment in practice. We combined PBM data on remdesivir distribution under the EUA with national VHA electronic records and administrative data to conduct a cohort study of the outcomes associated with remdesivir treatment among patients hospitalized with COVID-19. Our primary objective was to assess the association between remdesivir receipt and all-cause 30-day mortality. We also examined associations between remdesivir use and time to hospital discharge with in-hospital death as a competing event.

## Methods

This was a retrospective cohort study of patients with laboratory-confirmed COVID-19 with a first admission to acute care settings in VHA hospitals between May 1 and October 8, 2020. The institutional review board at the University of Iowa approved all data analyses and granted a waiver of informed consent per its policy for large analyses of secondary data generated during routine health care delivery. The study followed the Strengthening the Reporting of Observational Studies in Epidemiology (STROBE) reporting guideline.^15^

### Data Sources and Study Cohort

We obtained data from 3 sources: (1) the VHA Corporate Data Warehouse, which contains data on acute care stays, outpatient visits, inpatient and outpatient diagnoses by *International Statistical Classification of Diseases, Tenth Revision, Clinical Modification *(*ICD-10-CM*) codes, laboratory values, vital signs, prescribed outpatient and inpatient medications, and day of death in hospital and community settings; (2) the VHA COVID-19 Shared Data Resource, which contains variables for fact and day of initiation of mechanical ventilation in VHA hospitals (eMethods in the Supplement)^16^; and (3) the PBM remdesivir emergency use data file, which contains data on remdesivir shipment and administration during the EUA. We used the PBM data to validate VHA Corporate Data Warehouse medication administration data for remdesivir during emergency use.

**Figure 1.**
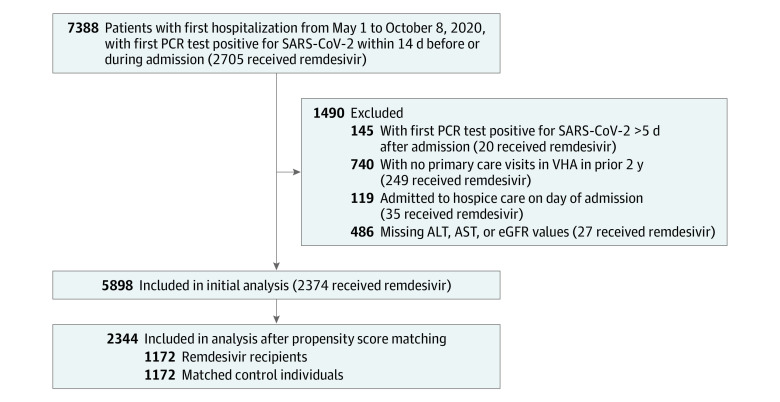
Cohort Derivation Flowchart ALT indicates alanine aminotransferase; AST, aspartate aminotransferase; eGFR, estimated glomerular filtration rate; PCR, polymerase chain reaction; and VHA, Veterans Health Administration.

We first identified all 7388 patients with a first admission to a VHA acute care setting between May 1 and October 8, 2020 (ie, during the remdesivir EUA), with a first polymerase chain reaction (PCR) test positive for SARS-CoV-2 within 14 days before or during hospitalization excluding readmissions (Figure 1). We excluded (1) 145 patients with a first positive PCR test result more than 5 days after admission because these patients may have acquired COVID-19 in the hospital; (2) 740 patients with no primary care visits to the VHA in the 2 years before admission because they lacked data on comorbidity and other risk adjustment variables; (3) 119 patients admitted to hospice care in the inpatient setting on the first day of hospitalization; and (4) 486 patients with no valid values for alanine aminotransferase (ALT), aspartate aminotransferase (AST), or estimated glomerular filtration rate (eGFR) during the hospital stay because PBM limited remdesivir availability to patients with ALT and AST values less than 5 times the upper limit of normal and an eGFR greater than 30 mL/min/1.73 m^3^. This left 5898 patients in the initial study cohort. We then used propensity score matching to create a second, analytic cohort of patients receiving and not receiving remdesivir.

### Variables

The exposure was remdesivir receipt as a time-dependent variable by hospital day. Outcomes were time to all-cause mortality within 30 days of remdesivir initiation (or 30 days of the corresponding hospital day at the time of matching for controls) and time to hospital discharge. Risk adjustment variables included patient age, sex, race/ethnicity, comorbidity, time of positive PCR test result relative to admission, mechanical ventilation use, intensive care unit (ICU) admission, laboratory values (ALT, AST, serum creatinine, eGFR, and total white blood cell count), vital signs (temperature, blood pressure, respiratory rate, and arterial oxygen saturation), outpatient medications before admission, inpatient mediations, and admission month. Race and ethnicity were recorded in the medical record at each medical encounter through patient self-report or patient-proxy report and were included in analyses to assess inclusivity and avoid confounding. We classified ICU stays, first day of mechanical ventilation, laboratory values, vital signs, and inpatient medications as time-dependent variables by hospital day, choosing the most extreme value for laboratory values and vital signs if there was more than 1 recorded in a day. We classified comorbidities based on inpatient and outpatient *ICD-10-CM* codes in the VHA Corporate Data Warehouse in the 2 years before admission using the method of Quan et al^17^ (eTable 1 in the Supplement). We did not have data on the amount of supplemental oxygen that patients required on each hospital day.

### Statistical Analysis

We used 2 methods to address confounding by indication and the time-dependent nature of treatment and illness severity. Our primary method involved propensity score matching of patients initiating remdesivir treatment to control patients who had not initiated remdesivir treatment by the same hospital day. To ensure consistency of the results, we also applied an alternate approach using marginal structural models with inverse probability of treatment weights by hospital day, following the method of Hernán et al and Robins et al,^18,19^ and compared the findings.

The matching strategy using propensity scores by hospital day is described in detail in eFigure 1 in the Supplement. In brief, we created a separate record for each day of acute care stay for each patient until the patient was discharged, became ineligible for remdesivir initiation owing to elevated ALT or AST values or an eGFR less than 30 mL/min/1.73 m^3^, or initiated remdesivir. Because patients with COVID-19 were often treated with both remdesivir and dexamethasone,^20^ we separated patient days according to inpatient dexamethasone use on or before each day. We then estimated separate logistic regression models to assess the likelihood of remdesivir treatment initiation on each hospital day among patients stratified by dexamethasone treatment. Candidate variables for propensity models were chosen based on literature review and clinical experience and included baseline demographic characteristics, comorbidities, prior outpatient medication use, admission month, and a series of time-dependent variables by hospital day, including laboratory values, vital signs, inpatient medications, mechanical ventilation use, and ICU stay up to and including the hospital day at the time of matching.

Each patient who initiated remdesivir treatment on a given hospital day was matched to a similar patient who had not initiated remdesivir treatment by the same hospital day. The day of matching was then defined as the index day for the outcome observation for both the remdesivir recipient and the control individual. Control patients who initiated remdesivir treatment on a later day during hospitalization were censored from follow-up at the time of remdesivir treatment initiation. To evaluate the quality of matching, we calculated standardized differences between characteristics of matched remdesivir recipients and controls as of the index day of matching, where standardized differences less than 10% suggested good covariate balance.^21^ We used Cox proportional hazards regression models to estimate differences in treatment outcomes in the matched cohort, censoring control patients who later initiated remdesivir treatment at the time of initiation and all patients who had not died at 30 days. Models included a random effect for hospitals and further controlled for residual differences in patient characteristics after matching (ie, age, race/ethnicity, outpatient medications, comorbidity, vital signs, ICU status and mechanical ventilation use on the day of matching, and the calendar day of matching). We report 2-sided *P* values using a significance threshold of .05.

For analyses of time from matching to hospital discharge with death as a competing risk, we generated cumulative incidence function plots for discharge among remdesivir recipients and controls and estimated Fine-Gray subdistribution HRs for discharge.^22^ To explore potential associations between completion of remdesivir treatment courses and time of hospital discharge, we plotted days from matching to discharge among remdesivir recipients and controls as well as the total number of days that remdesivir treatment was received in the hospital among remdesivir recipients. These plots excluded patients who died before discharge.

We describe our alternate approach to data analyses using marginal structural models in the eMethods in the Supplement. In brief, we began with the entire study cohort and weighted the contribution of each patient on a given hospital day using stabilized weights incorporating baseline and time-varying patient covariates.^18,19^ Associations between remdesivir use and outcomes (ie, time to mortality and hospital discharge) were then assessed using weighted pooled models (eMethods in the Supplement). All data were analyzed using SAS, version 9.4 (SAS Institute Inc).

## Results

### Initial Cohort Before Matching

The initial cohort included 5898 patients admitted to 123 hospitals, 2374 (40.3%) of whom received remdesivir treatment (2238 men [94.3%]) (Table 1). Compared with patients who never received remdesivir treatment during hospitalization (3302 men [93.7%]), remdesivir recipients were older (mean [SD] age, 67.8 [12.8] years vs 67.0 [14.4] years; *P* = .03), more likely to be White (1414 patients [59.6%] vs 1916 [54.4%]; *P* < .001), more likely to have chronic obstructive pulmonary disease (889 patients [37.4%] vs 1127 [32.0%]; *P* < .001), and more ill at admission based on ICU care and vital signs.

**Table 1.** Patient Characteristics by Remdesivir Receipt During Hospitalization

Characteristic	Patients (N = 5898)^a^		Standardized difference, %
	Received remdesivir	Did not receive remdesivir	
Total patients	2374 (40.3)	3524 (59.7)	NA
Age, mean (SD), y	67.8 (12.8)	67.0 (14.4)	5.7
Sex			
Male	2238 (94.3)	3302 (93.7)	4.6
Female	136 (5.7)	222 (6.3)	−2.3
Race/ethnicity			
White	1414 (59.6)	1916 (54.4)	10.5
Black	745 (31.4)	1330 (37.7)	−13.4
Other^b^	70 (3.0)	92 (2.6)	2.4
Missing	145 (6.0)	186 (5.3)	3.6
Admission month			
May	184 (7.8)	558 (15.8)	−25.3
June	368 (15.5)	633 (18.0)	−6.6
July	866 (36.5)	1155 (32.8)	7.8
August	519 (21.9)	694 (19.7)	5.4
September or October	437 (18.4)	484 (13.7)	12.8
Comorbidity			
Myocardial infarction	233 (9.8)	461 (13.1)	−10.7
Congestive heart failure	550 (23.2)	955 (27.1)	−9.1
Peripheral vascular disease	421 (17.7)	782 (22.2)	−11.2
Cerebrovascular disease	368 (15.5)	722 (20.5)	−13.0
Arrhythmia	1075 (45.3)	1570 (44.6)	1.5
Hypertension	2010 (84.7)	2926 (83.0)	4.5
Diabetes	1330 (56.0)	1792 (50.9)	10.4
Chronic obstructive pulmonary disease	889 (37.4)	1127 (32.0)	11.5
Kidney disease	694 (29.2)	1265 (35.9)	−14.3
Cancer	376 (15.8)	573 (16.3)	−1.2
Liver disease	367 (15.5)	624 (17.7)	−6.1
Dementia	328 (13.8)	652 (18.5)	−12.8
Obesity	1080 (45.5)	1266 (35.9)	19.6
Alcohol diagnosis	271 (11.4)	681 (19.3)	−22.1
Drug use diagnosis	201 (8.5)	536 (15.2)	−21.0
Oxygen saturation, mean (SD), %	90.6 (8.2)	94.1 (6.1)	−46.5
Oxygen saturation <94%	1822 (80.4)	1612 (46.9)	74.2
Temperature, mean (SD), °C	37.5 (1.7)	37.1 (0.7)	28.1
BP, mean (SD), mm Hg			
Systolic	118.3 (18.6)	119.3 (20.6)	−5.2
Diastolic	67.0 (11.3)	67.7 (12.3)	−5.4
Respiratory rate, mean (SD), breaths/min	23.9 (7.2)	21.0 (5.1)	45.3
WBC count, mean (SD), 10^9^ cells/L	7.3 (3.8)	7.1 (3.9)	5.1
eGFR, mean (SD), mL/min/1.73 m^3^	65.3 (23.0)	63.6 (29.4)	6.5
eGFR <30 mL/min/1.73 m^3^	130 (5.7)	561 (16.0)	–33.6
AST level, mean (SD), U/L	50.6 (55.5)	44.3 (123.8)	6.6
ALT level, mean (SD), U/L	40.0 (46.3)	38.8 (118.9)	1.6
Positive PCR test result at or before admission	2357 (99.3)	3450 (97.9)	10.5
ICU care at admission	529 (22.3)	459 (13.0)	24.6
Medications before admission			
Systemic corticosteroid	107 (4.5)	104 (3.0)	8.2
Azithromycin	90 (3.8)	76 (2.2)	9.6
Other antibiotic	153 (6.4)	168 (4.8)	7.3
Hydroxychloroquine or chloroquine	14 (0.6)	11 (0.3)	4.1
Statin	1183 (49.8)	1465 (41.6)	16.6
ACE inhibitor	604 (25.4)	724 (20.5)	11.7
ARB	320 (13.5)	426 (12.1)	4.2
Warfarin or direct oral anticoagulant	240 (10.1)	405 (11.5)	−4.5
Famotidine	91 (3.8)	117 (3.3)	2.8
Medications during admission			
Dexamethasone	1893 (79.9)	778 (22.1)	140.4
Other systemic corticosteroid	356 (15.0)	296 (8.4)	20.3
Azithromycin	909 (38.3)	698 (19.8)	41.4
Other antibiotic	1384 (58.2)	1347 (38.2)	40.6
Hydroxychloroquine or chloroquine	17 (0.7)	48 (1.4)	−6.6
Statin	1371 (57.7)	1862 (52.8)	9.4
ACE inhibitor	534 (22.5)	664 (18.8)	8.9
ARB	305 (12.9)	428 (12.1)	2.1
Heparin	651 (27.4)	935 (26.5)	1.9
Low-molecular-weight heparin	1921 (80.8)	2035 (57.7)	51.2
Warfarin or direct oral anticoagulant	441 (18.6)	693 (19.7)	−2.8
Famotidine	447 (18.8)	392 (11.1)	21.4
Care during admission			
ICU stay	714 (30.1)	652 (18.5)	51.6
Mechanical ventilation	187 (7.9)	173 (4.9)	44.6
Outcomes			
Death within 30 d	377 (15.9)	338 (9.6)	NA
Length of stay, median (IQR), d	8.0 (5-15)	4.0 (2-9)	NA

Abbreviations: ACE, angiotensin-converting enzyme; ALT, alanine aminotransferase; ARB, angiotensin receptor blocker; AST, aspartate aminotransferase; BP, blood pressure; eGFR, estimated glomerular filtration rate; ICU, intensive care unit; IQR, interquartile range; NA, not applicable; PCR, polymerase chain reaction; WBC, white blood cell.

SI conversion factor: To convert AST and ALT levels to μkat/L, multiply by 0.0167.

^a^ Data are presented as number (percentage) of patients unless otherwise indicated.

^b^ Other race/ethnicity includes American Indian or Alaska Native, Asian, and Native Hawaiian or other Pacific Islander.

### Propensity Score–Matched Cohort

We were able to match each of 1172 patients initiating remdesivir to a control patient on the same hospital day, yielding a final matched cohort of 2344 individuals (Table 2). Remdesivir recipients and matched controls were similar with regard to age (mean [SD], 66.6 [14.2] years vs 67.5 [14.1] years) and sex (1101 men [93.9%] vs 1101 men [93.9%]). The matched cohort included 559 remdesivir recipients who had also received dexamethasone treatment and 613 remdesivir recipients who had not received dexamethasone matched to identical numbers of controls who had and had not received dexamethasone treatment. The 1172 matched remdesivir recipients represented 58.3% of the 2011 patients who had complete data on the day of remdesivir treatment initiation and were eligible for matching. Compared with the 1172 remdesivir recipients in the matched cohort, the 839 remdesivir recipients with complete data who could not be matched had a greater propensity for remdesivir treatment and indications of greater illness severity (eFigure 2 and eTable 2 in the Supplement).

**Table 2.** Patient Characteristics in the Propensity Score–Matched Cohort

Characteristic	Patients (N = 2344)^a^		Standardized difference, %
	Remdesivir recipients (n = 1172)	Controls (n = 1172)	
Age, mean (SD), y	66.6 (14.2)	67.5 (14.1)	−6.73
Age, y			
<55	228 (19.5)	197 (16.8)	7.13
55-64	241 (20.6)	234 (20.0)	1.48
65-74	387 (33.0)	405 (34.6)	−3.41
75-84	202 (17.2)	197 (16.8)	0.90
>84	114 (9.7)	139 (11.8)	−6.93
Sex			
Male	1101 (93.9)	1101 (93.9)	0
Female	71 (6.1)	71 (6.1)	0
Race/ethnicity			
White	693 (59.1)	674 (57.5)	3.38
Black	388 (33.1)	406 (34.6)	−3.06
Other^b^	27 (2.3)	38 (3.2)	−5.39
Missing	65 (5.5)	55 (4.7)	3.37
Admission month			
May	124 (10.6)	151 (12.9)	−7.29
June	230 (19.6)	213 (18.2)	3.90
July	428 (36.5)	391 (33.4)	6.62
August	198 (16.9)	238 (20.3)	−8.42
September or October	192 (16.4)	179 (15.3)	3.02
Positive PCR test result at or before admission	1160 (99.0)	1154 (98.5)	4.29
Hospital day at matching			
1	309 (26.4)	309 (26.4)	0
2	393 (33.5)	393 (33.5)	0
3	207 (17.7)	207 (17.7)	0
4 or 5	157 (13.4)	157 (13.4)	0
6-8	73 (6.2)	73 (6.2)	0
9	33 (2.9)	33 (2.9)	0
Comorbidity			
Myocardial infarction	105 (9.0)	121 (10.3)	−4.42
Congestive heart failure	257 (21.9)	267 (22.8)	−2.06
Peripheral vascular disease	214 (18.3)	236 (20.1)	−4.82
Cerebrovascular disease	166 (14.2)	189 (16.1)	−5.24
Arrhythmia	522 (44.5)	495 (42.2)	4.64
Hypertension	959 (81.8)	970 (82.8)	−2.68
Diabetes	627 (53.5)	573 (48.9)	9.24
Chronic obstructive pulmonary disease	405 (34.6)	422 (36.0)	−2.87
Kidney disease	298 (25.4)	308 (26.3)	−1.94
Cancer	157 (13.4)	168 (14.3)	−2.34
Liver disease	195 (16.6)	188 (16.0)	1.62
Dementia	170 (14.5)	180 (15.4)	−2.57
Obesity	518 (44.2)	513 (43.8)	0.87
Alcohol use diagnosis	154 (13.1)	189 (16.1)	−8.20
Drug use diagnosis	116 (9.9)	134 (11.4)	−4.46
Care at matching			
ICU stay	242 (20.7)	234 (19.1)	4.42
Mechanical ventilation	69 (5.9)	45 (3.8)	9.75
Laboratory value at matching			
Oxygen saturation, mean (SD), %	91.4 (5.4)	91.8 (5.6)	−8.12
Oxygen saturation <94%	954 (81.4)	954 (81.4)	0
Oxygen saturation <94% ever before matching	1048 (89.5)	1026 (87.6)	4.85
Temperature, mean (SD), °C	37.5 (2.0)	37.5 (0.8)	0
BP, mean (SD), mm Hg			
Systolic	117.9 (16.8)	118.5 (17.7)	−3.50
Diastolic	66.9 (10.3)	67.3 (11.2)	−3.79
Respiratory rate, mean (SD), breaths/min	22.9 (6.7)	22.3 (6.2)	9.30
WBC count, mean (SD), 10^9^ cells/L	7.3 (3.9)	7.1 (3.8)	4.40
eGFR, mean (SD), mL/min/1.73 m^3^	74.5 (21.7)	74.6 (22.1)	−0.47
AST level, mean (SD), U/L	46.7 (32.5)	44.2 (34.9)	8.06
ALT level, mean (SD), U/L	39.6 (32.7)	38.2 (33.9)	2.59
Medication at matching			
Dexamethasone	559 (47.7)	559 (47.7)	0
Any corticosteroid	627 (53.5)	616 (52.6)	2.17
Azithromycin	280 (23.9)	268 (22.9)	2.77
Other antibiotic	406 (34.6)	413 (35.2)	−1.54
Heparin	165 (14.1)	161 (13.7)	1.27
Low-molecular-weight heparin	721 (61.5)	723 (61.7)	−0.35
Warfarin or direct oral anticoagulant	120 (10.2)	135 (11.5)	−4.29
Famotidine	122 (10.4)	104 (8.9)	5.39
Statin	496 (42.3)	533 (45.5)	–6.42
ACE inhibitor	129 (11.0)	158 (13.5)	−7.31
ARB	79 (6.7)	102 (8.7)	−7.73
Hydroxychloroquine or chloroquine	6 (0.5)	5 (0.4)	1.09
Medication before admission			
Warfarin or direct oral anticoagulant	114 (9.7)	128 (10.9)	−3.91
Famotidine	47 (4.0)	47 (4.0)	0.
Statin	544 (46.4)	554 (47.2)	−1.55
ACE inhibitor	270 (23.0)	271 (23.1)	−0.20
ARB	136 (11.6)	154 (13.1)	−4.68
Hydroxychloroquine or chloroquine	9 (0.8)	4 (0.3)	6.47

Abbreviations: ACE, angiotensin-converting enzyme; ALT, alanine aminotransferase; ARB, angiotensin receptor blocker; AST, aspartate aminotransferase; BP, blood pressure; eGFR, estimated glomerular filtration rate; ICU, intensive care unit; PCR, polymerase chain reaction; WBC, white blood cell.

SI conversion factor: To convert AST and ALT levels to μkat/L, multiply by 0.0167.

^a^ Data are presented as number (percentage) of patients unless otherwise indicated. All values are from the day of matching (ie, day of remdesivir initiation or corresponding hospital day for controls).

^b^ Other race/ethnicity includes American Indian or Alaska Native, Asian, and Native Hawaiian or other Pacific Islander.

Remdesivir recipients and controls in the matched cohort were similar with regard to race/ethnicity, comorbidities, month of admission, illness severity on the hospital day of matching (as indicated by vital signs), and laboratory values (Table 2). The proportions of remdesivir recipients and controls who were admitted to the ICU (242 patients [20.7%] vs 234 [19.1%]) and received mechanical ventilation (69 patients [5.9%] vs 45 [3.8%]) were also similar. The same proportion of remdesivir recipients and controls had an oxygen saturation level less than 94%, a commonly recommended threshold for remdesivir treatment,^10^ on the day of matching (954 patients [81.4%] in each group). Similar proportions had ever had an oxygen saturation level less than 94% at any point during hospitalization before matching (1048 [89.5%] vs 1026 [87.6%]).^10^ Similar proportions of remdesivir recipients and controls received dexamethasone treatment before the day of matching (551 [47.0%] vs 545 [46.5%]), and the same proportion in each group received dexamethasone treatment on the day of matching (559 [47.7%]). Standardized differences were less than 10% for all measures (Table 2).

### Outcomes

**Figure 2.**
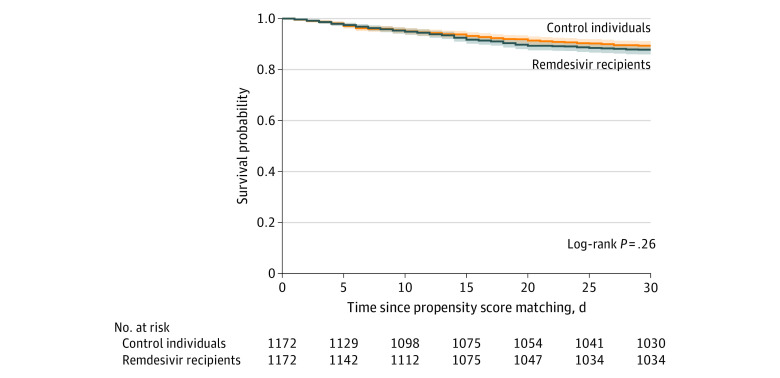
Kaplan-Meier Survival Curves for Remdesivir Recipients and Control Individuals in the Propensity Score–Matched Cohort Day 0 is the day of matching (ie, day of remdesivir initiation or corresponding hospital day for controls).

A total of 267 patients (11.4%) died within 30 days of the day of matching, including 143 remdesivir recipients (12.2%) and 124 controls (10.6%) (log rank *P* = .26 for the difference in Kaplan-Meier survival curves) (Figure 2). Seventy control patients (6%) were censored at a median of 4 days (interquartile range [IQR], 4-6 days) after matching when they initiated remdesivir treatment. Remdesivir recipients and controls had similar HRs for mortality within 30 days in Cox proportional hazards regression models (adjusted HR, 1.06; 95% CI, 0.83-1.36). Mortality at 30 days was also similar among subgroups of patients receiving and not receiving dexamethasone treatment at remdesivir initiation (dexamethasone recipients: adjusted HR, 0.93; 95% CI, 0.64-1.35; nonrecipients: adjusted HR, 1.19; 95% CI, 0.84-1.69) (Kaplan-Meier survival curves are shown in eFigures 3 and 4 in the Supplement). Results were similar in a sensitivity analysis that compared patients who initiated remdesivir treatment within 48 hours of admission with matched controls who did not initiate remdesivir treatment within 48 hours (adjusted HR, 0.95; 95% CI, 0.82-1.10).

**Figure 3.**
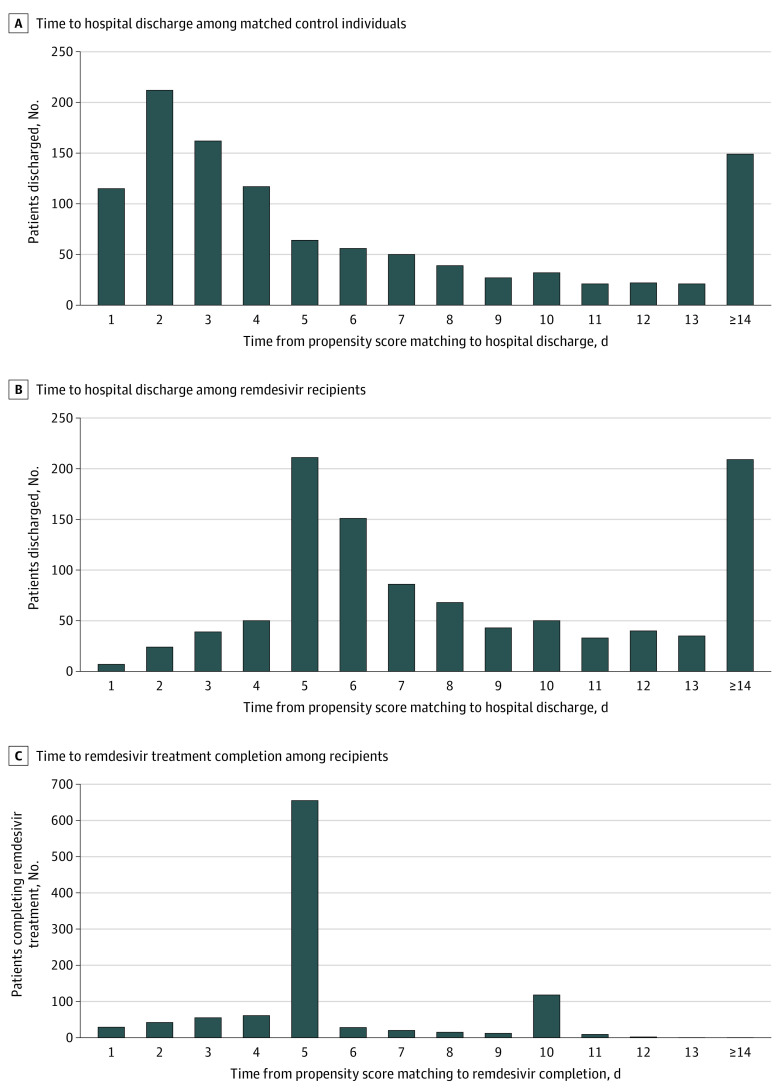
Distribution of Days to Remdesivir Treatment Completion Among Recipients and Days to Hospital Discharge Among Recipients and Controls

Remdesivir recipients had a longer median time to hospital discharge after matching (6 days [IQR, 4-12 days]) compared with controls (3 days [IQR, 1-7 days]) (*P* < .001 for comparison). Cumulative incidence function plots for hospital discharge (eFigure 5 in the Supplement) indicated delayed discharge after matching among remdesivir recipients compared with controls (Gray test for inequality, <0.01). The Fine-Gray subdistribution HR for time to discharge for remdesivir recipients compared with controls was 0.65 (95% CI, 0.60-0.71). Most of the remdesivir recipients who survived to discharge (773 [73.9%]) received a full 5-day or 10-day course of remdesivir while hospitalized. The distribution of days from matching to hospital discharge showed a larger number of discharges from days 1 to 4 among controls compared with a larger number of discharges on days 5 and 6 among remdesivir recipients in association with a large number of patients completing a remdesivir course on day 5 (Figure 3).

Results were similar in analyses using marginal structural models. The HR for death within 30 days in these analyses was 0.98 (95% CI, 0.71-1.35) for remdesivir recipients compared with controls, and the HR for hospital discharge was 0.72 (95% CI, 0.53-0.97).

## Discussion

In this cohort study of US veterans hospitalized with COVID-19 at Veterans Affairs facilities, remdesivir treatment was associated with prolonged hospitalization but was not associated with improved survival. The finding of a longer time to hospital discharge in association with remdesivir treatment represents a potential unintended and undesirable consequence of remdesivir adoption in practice. If remdesivir use shortened time to recovery from COVID-19, as indicated by the ACTT-1,^4^ hospital stays could be shorter and more beds could become available to treat more patients during COVID-19 surges. This would be a substantial benefit during a pandemic that is straining hospital resources regardless of any association with mortality. As other researchers have noted,^23^ the ACTT-1 excluded patients who were expected to be discharged within 72 hours; thus, it was difficult to extrapolate ACTT-1 study findings to length of hospital stay among patients treated with remdesivir in routine practice. The current study suggests that remdesivir treatment was associated with an increased time to hospital discharge as it was administered in routine clinical settings.

Why would remdesivir treatment extend length of stay? Complications of treatment, such as kidney injury, could extend hospitalizations, but rates of adverse events associated with remdesivir were low in trials.^4^ It is also possible that clinicians were not discharging patients who otherwise met the criteria for hospital discharge until the remdesivir course was completed. The recommended remdesivir treatment course is a somewhat arbitrary 5 or 10 days depending on illness severity, and remdesivir is currently available only as an intravenous formulation for use in health care settings.^10^ Treatment guidelines recommend against keeping patients in the hospital simply to complete a course of remdesivir treatment, but there are anecdotes of this occurring.^11,24^ Our examination of days from matching to hospital discharge showed a shift in discharges from days 1 to 4 among controls to day 5 or 6 among remdesivir recipients, in association with large numbers of patients completing 5-day remdesivir courses. These findings suggest that clinicians may have not discharged some patients who were receiving remdesivir until they completed a 5-day course. If this was the case, routine use of remdesivir for COVID-19 may have been associated with increased use of scarce hospital beds during the pandemic without being associated with improvements in patient survival.

### Limitations

This study has limitations. First, as in all observational studies, there is potential for unadjusted confounding associated with illness severity. Propensity score–matched remdesivir recipients and controls had similar illness severity based on observed variables, but there may have been residual confounding associated with both unobserved variables and imprecise measurement of observed variables. Residual differences in illness severity could obscure improvements in survival and may explain the longer length of hospital stay among remdesivir recipients compared with controls.

Second, the results pertain only to the 1172 remdesivir recipients (49.5% of the remdesivir recipients in the total cohort) whom we were able to match to controls. These patients had a lower propensity for remdesivir treatment and less severe illness compared with unmatched remdesivir recipients. Part of the reason was that rates of remdesivir treatment were high among the most severely ill patients, leaving few similar control patients for matching. This study’s findings should not be extrapolated to patients who do not resemble those in the propensity score–matched cohort. In addition, this study of US veterans included a small number of women, which affects the generalizability of the findings to the overall population.

Third, limitations in available data prevented us from identifying specific subgroups of patients who may have been more likely to benefit from remdesivir treatment and from precisely emulating clinical trials. Subgroup analyses in the ACTT-1 suggested that remdesivir was most effective when patients required supplemental oxygen but had not yet progressed to require mechanical ventilation.^4^ It is biologically plausible that remdesivir treatment is most beneficial during the early, viral replication phase of COVID-19, when antiviral drugs can still alter the course of illness before severe lung injury occurs.^3^ Although we had data on oxygen saturation levels for patients during hospitalization and the matched remdesivir recipients and controls were balanced based on these values, we lacked data on the time from symptom onset to remdesivir initiation and the amount of supplemental oxygen patients required during hospitalization. We were therefore not able to examine variation in the outcomes associated with remdesivir according to phase of illness.

## Conclusions

In this cohort study of US veterans hospitalized with COVID-19, remdesivir treatment was not associated with survival but was associated with longer hospitalization. These findings suggest that routine use of remdesivir may be associated with increased hospital bed use while not being associated with improvements in patient survival.

## Notes

Supplement. **eTable 1.** ICD-10 codes used to define comorbidities eMethods. **eFigure 1.** Propensity matching scheme **eFigure 2.** Distribution of propensity scores among unmatched and matched remdesivir recipients **eTable 2.** Characteristics of matched and unmatched remdesivir recipients **eFigure 3.** Kaplan-Meier survival curves for matched cohort with dexamethasone use at match **eFigure 4.** Kaplan-Meier survival curves for matched cohort without dexamethasone use at match **eFigure 5.** Cumulative incidence function plots for time to hospital discharge eReferences

## References

[1] Agostini ML, Andres EL, Sims AC, . Coronavirus susceptibility to the antiviral remdesivir (GS-5734) is mediated by the viral polymerase and the proofreading exoribonuclease. mBio. 2018;9(2):e00221-18. doi:10.1128/mBio.00221-18 29511076 PMC5844999

[2] Dolin R, Hirsch MS. Remdesivir—an important first step. N Engl J Med. 2020;383(19):1886-1887. doi:10.1056/NEJMe2018715 32459913 PMC7269011

[3] Harrington DP, Baden LR, Hogan JW. A large, simple trial leading to complex questions. N Engl J Med. 2021;384(6):576-577. doi:10.1056/NEJMe2034294 33264557 PMC7727323

[4] Beigel JH, Tomashek KM, Dodd LE, ; ACTT-1 Study Group Members. Remdesivir for the treatment of Covid-19—final report. N Engl J Med. 2020;383(19):1813-1826. doi:10.1056/NEJMoa2007764 32445440 PMC7262788

[5] Pan H, Peto R, Henao-Restrepo AM, ; WHO Solidarity Trial Consortium. Repurposed antiviral drugs for Covid-19—interim WHO Solidarity Trial results. N Engl J Med. 2021;384(6):497-511. doi:10.1056/NEJMoa2023184 33264556 PMC7727327

[6] Spinner CD, Gottlieb RL, Criner GJ, ; GS-US-540-5774 Investigators. Effect of remdesivir vs standard care on clinical status at 11 days in patients with moderate COVID-19: a randomized clinical trial. JAMA. 2020;324(11):1048-1057. doi:10.1001/jama.2020.16349 32821939 PMC7442954

[7] Wang Y, Zhang D, Du G, . Remdesivir in adults with severe COVID-19: a randomised, double-blind, placebo-controlled, multicentre trial. Lancet. 2020;395(10236):1569-1578. doi:10.1016/S0140-6736(20)31022-9 32423584 PMC7190303

[8] United States Food and Drug Administration. Remdesivir emergency use authorization letter. Accessed May 30, 2020. https://www.fda.gov/media/137564/download

[9] Rubin D, Chan-Tack K, Farley J, Sherwat A. FDA approval of remdesivir—a step in the right direction. N Engl J Med. 2020;383(27):2598-2600. doi:10.1056/NEJMp2032369 33264539

[10] Infectious Diseases Society of America. ISDA guidelines on the treatment and management of patients with COVID-19. Updated December 2, 2020. Accessed December 3, 2020. https://www.idsociety.org/practice-guideline/covid-19-guideline-treatment-and-management/#toc-8

[11] National Institutes of Health. Coronavirus disease 2019 (COVID-19) treatment guidelines. Accessed December 3, 2020. https://www.covid19treatmentguidelines.nih.gov/34003615

[12] World Health Organization. Therapeutics and COVID-19: living guideline. Updated November 20, 2020. Accessed December 3, 2020. https://www.who.int/publications/i/item/therapeutics-and-covid-19-living-guideline

[13] US Department of Veterans Affairs. Veterans Health Administration. Accessed December 6, 2020. https://www.va.gov/health/

[14] VA Pharmacy Benefits Management Services. Remdesivir emergency use authorization (EUA) requirements May 2020. Accessed June 18, 2020. https://www.va.gov/covidtraining/docs/20200618_Dynamic_Drugs_in_the_Battle_of_COVID_19/Remdesivir_Emergency_Use_Authorization_Requirements.pdf

[15] von Elm E, Altman DG, Egger M, Pocock SJ, Gøtzsche PC, Vandenbroucke JP; STROBE Initiative. The Strengthening the Reporting of Observational Studies in Epidemiology (STROBE) statement: guidelines for reporting observational studies. Lancet. 2007;370(9596):1453-1457. doi:10.1016/S0140-6736(07)61602-X 18064739

[16] VA Informatics and Computing Infrastructure. VA COVID-19 shared data resource: update. US Department of Veterans Affairs. Accessed May 29, 2021. https://www.hsrd.research.va.gov/for_researchers/cyber_seminars/archives/3834-notes.pdf

[17] Quan H, Sundararajan V, Halfon P, . Coding algorithms for defining comorbidities in ICD-9-CM and ICD-10 administrative data. Med Care. 2005;43(11):1130-1139. doi:10.1097/01.mlr.0000182534.19832.83 16224307

[18] Hernán MA, Brumback B, Robins JM. Marginal structural models to estimate the causal effect of zidovudine on the survival of HIV-positive men. Epidemiology. 2000;11(5):561-570. doi:10.1097/00001648-200009000-00012 10955409

[19] Robins JM, Hernán MA, Brumback B. Marginal structural models and causal inference in epidemiology. Epidemiology. 2000;11(5):550-560. doi:10.1097/00001648-200009000-00011 10955408

[20] Horby P, Lim WS, Emberson JR, et al; RECOVERY Collaborative Group. Dexamethasone in hospitalized patients with COVID-19—preliminary report. N Engl J Med. 2021;384(8):693-704. doi:10.1056/NEJMoa2021436 32678530 PMC7383595

[21] Austin PC. Balance diagnostics for comparing the distribution of baseline covariates between treatment groups in propensity-score matched samples. Stat Med. 2009;28(25):3083-3107. doi:10.1002/sim.3697 19757444 PMC3472075

[22] Austin PC, Fine JP. Practical recommendations for reporting Fine-Gray model analyses for competing risk data. Stat Med. 2017;36(27):4391-4400. doi:10.1002/sim.7501 28913837 PMC5698744

[23] Anderson MR, Bach PB, Baldwin MR. Hospital length of stay for patients with severe COVID-19: implications for remdesivir’s value. *Pharmacoecon Open*. 2021;5(1):129-131. doi:10.1007/s41669-020-00243-6 PMC7734451 33315210

[24] Griffin D, Racaniello V. COVID-19 clinical update #41 with Dr Daniel Griffin. *This Week in Virology*. December 18, 2020. Accessed December 26, 2020. https://www.microbe.tv/twiv/twiv-695/

