# Supplementary material for: Association of Remdesivir Treatment With Survival and Length of Hospital Stay Among US Veterans Hospitalized With COVID-19
Source: JAMA Netw Open. 2021 Jul 15;4(7):e2114741. doi: 10.1001/jamanetworkopen.2021.14741 (PMC8283561; doi:10.1001/jamanetworkopen.2021.14741)
Supplement: Supplement. — eTable 1. ICD-10 codes used to define comorbidities eMethods. eFigure 1. Propensity matching scheme eFigure 2. Distribution of propensity scores among unmatched and matched remdesivir recipients eTable 2. Characteristics of matched and unmatched remdesivir recipients eFigure 3. Kaplan-Meier survival curves for matched cohort with dexamethasone use at match eFigure 4. Kaplan-Meier survival curves for matched cohort without dexamethasone use at match eFigure 5. Cumulative incidence function plots for time to hospital discharge eReferences [file jamanetwopen-e2114741-s001.pdf]

Supplementary Online Content

Ohl ME, Miller DR, Lund BC, et al. Association of remdesivir treatment with survival and length of hospital stay among US veterans hospitalized with COVID-19. *JAMA Netw Open*. 2021;4(7):e2114741. doi:10.1001/jamanetworkopen.2021.14741

eTable 1. ICD-10 codes used to define comorbidities

eMethods.

eFigure 1. Propensity matching scheme

eFigure 2. Distribution of propensity scores among unmatched and matched remdesivir recipients

eTable 2. Characteristics of matched and unmatched remdesivir recipients

eFigure 3. Kaplan-Meier survival curves for matched cohort with dexamethasone use at match

eFigure 4. Kaplan-Meier survival curves for matched cohort without dexamethasone use at match

eFigure 5. Cumulative incidence function plots for time to hospital discharge

eReferences

This supplementary material has been provided by the authors to give readers additional information about their work.

© 2021 Ohl ME et al. *JAMA Network Open*.

eTable 1: ICD-10 codes used to define comorbidities following method of Quan[1]

Diagnosis	ICD10Codes
Alcohol diagnosis	E52.X, F10.X, G62.1, I42.6, K29.2, K70.0, K70.3, K70.9, T51.X, Z50.2, Z71.4, Z72.1
Arrhythmia	I44.1, I44.2, I44.3, I45.6, I45.9, I47.X, I48.X, I49.X, R00.0, R00.1, R00.8, T82.1, Z45.0, Z95.0
Cerebrovascular disease	G45.X, G46.X, H34.0, I60.X, I61.X, I62.X, I63.X, I64.X, I65.X, I66.X, I67.X, I68.X, I69.X
Congestive heart failure	I09.9, I11.0, I13.0, I13.2, I25.5, I42.0, I42.5, I42.6, I42.7, I42.8, I42.9, I43.x, I50.x, P29.0
Dementia	F00.X, F01.X, F02.X, F03.X, F05.1, G30.X, G31.1
Diabetes	E10.0, E10.1, E10.2, E10.3, E10.4, E10.5, E10.6, E10.7, E10.8, E10.9, E11.0, E11.1, E11.2, E11.3, E11.4, E11.5, E11.6, E11.7, E11.8, E11.9, E12.0, E12.1, E12.2, E12.3, E12.4, E12.5, E12.6, E12.7, E12.8, E12.9, E13.0, E13.1, E13.2, E13.3, E13.4, E13.5, E13.6, E13.7, E13.8, E13.9, E14.0, E14.1, E14.2, E14.3, E14.4, E14.5, E14.6, E14.7, E14.8, E14.9
Drug use diagnosis	F11.X, F12.X, F13.X, F14.X, F15.X, F16.X, F18.X, F19.X, Z71.5, Z72.2
Hypertension	I10.X, I11.X, I12.X, I13.X, I15.X
Liver disease	B18.X, I85.X, I86.4, I98.2, K70.X, K71.1, K71.3, K71.4, K71.5, K71.7, K72.X, K73.X, K74.X, K76.0, K76.2, K76.3, K76.4, K76.5, K76.6, K76.7, K76.8, K76.9, Z94.4
Malignancy	C01.X, C02.X, C03.X, C04.X, C05.X, C06.X, C07.X, C08.X, C09.X, C10.X, C11.X, C12.X, C13.X, C14.X, C15.X, C16.X, C17.X, C18.X, C19.X, C20.X, C21.X, C22.X, C23.X, C24.X, C25.X, C26.X, C30.X, C31.X, C32.X, C33.X, C34.X, C37.X, C38.X, C39.X, C40.X, C41.X, C43.X, C45.X, C46.X, C47.X, C48.X, C49.X, C50.X, C51.X, C52.X, C53.X, C54.X, C55.X, C56.X, C57.X, C58.X, C60.X, C61.X, C62.X, C63.X, C64.X, C65.X, C66.X, C67.X, C68.X, C69.X, C70.X, C71.X, C72.X, C73.X, C74.X, C75.X, C76.X, C81.X, C82.X, C83.X, C84.X, C85.X, C88.X, C90.X, C91.X, C92.X, C93.X, C94.X, C95.X, C96.X, C97.X
Myocardial infarction	I21.x, I22.x, I25.2
Obesity	E66.X
Peripheral vascular disease	I70.x, I71.x, I73.1, I73.8, I73.9, I77.1, I79.0, I79.2, K55.1, K55.8, K55.9, Z95.8, Z95.9
Renal disease	I12.0, I13.1, N03.2, N03.3, N03.4, N03.5, N03.6, N03.7, N05.2, N05.3, N05.4, N05.5, N05.6, N05.7, N18.X, N19.X, N25.0, Z49.0, Z49.1, Z49.2, Z94.0, Z99.2

eMethods

Extraction of data elements

Data elements were extracted from VHA's Corporate Data Warehouse (CDW) and variables defined following protocols used to create The COVID-19 Shared Data Resource (CSDR).[2] Data

© 2021 Ohl ME et al. *JAMA Network Open*.

extraction methods are available at

(https://vhacdwdwhweb100.vha.med.va.gov/phenotype/index.php/COVID-19:Shared_Data_Resource).

Variables for fact of ventilation and day of ventilation were created based on procedure codes, medications used in intubation and ventilation, and natural language processing of note text. Day of ventilation was validated by chart review

Approach to propensity score matching by hospital day

To estimate propensity models for remdesivir initiation, a separate record was created for each day of acute stay for each patient until the patient was discharged, became ineligible for remdesivir initiation due to elevated ALT/AST or eGFR<30, or initiated remdesivir. In addition, days of care on which key predictors of remdesivir were missing were excluded (e.g., missing eGFR). This resulted in 25,823 total patient days of care, of which 2011 represented days of initial remdesivir administration. Because dexamethasone was often used by patients who also receive remdesivir, we stratified patient days according to dexamethasone use, where dexamethasone patient-days were defined as days on which patients had current or previous dexamethasone during the hospitalization (n=7128 patient days with 1299 initial remdesivir), while non-dexamethasone patient-days were defined as days with no current or previous dexamethasone use (18695 patient-days with 712 representing initial remdesivir). Separate logistic regression models were then estimated to predict the likelihood of remdesivir initiation with and without current or prior dexamethasone use. The dependent variable for logistic regression models was defined to represent the day of initial remdesivir use. Candidate predictors for propensity models were identified based on literature review, clinical judgement, and a putative causal structure of the data, and included baseline demographics, comorbidities, prior outpatient medication use. Time-dependent variables included day of stay, laboratory values and vital signs recorded on the specific day, and inpatient medications, ventilation, and ICU stays up to and including the day represented by the patient-day observation. The main source of missing data involved laboratory and vital signs assessments by day of stay. To

© 2021 Ohl ME et al. *JAMA Network Open*.

address missingness for those variables, we first extrapolated past values up to two days forward. For example, serum creatinine on the first hospital day would be assigned to the second and third days, if creatinine was missing for Days 2 and 3. Due to the potential for bias when extrapolating measures too far from the measurement date, we limited the extrapolation to two days. Subsequently, patient days of stay that still had missing vitals and laboratory measures were excluded from propensity models and subsequent matching eligibility. The final cohort included the original 5,898 eligible patients and 58,517 patient-days without missing values.

Each patient who initiated remdesivir (i.e., “cases”) during the observation period was matched to a patient who had not initiated remdesivir as of the same day of stay (i.e., “controls”). The matching algorithm incorporated a novel approach using the nearest available Mahalanobis metric within calipers representing one quarter of one standard deviation of the case propensity score.[3] This matching algorithm produces better patient covariate balance compared to nearest neighbor propensity matching or matching by mahalanobis distance calculated using only the propensity score.[1]

Steps in the matching procedure are depicted in eFigure 1 below and include: 1) Identify a single randomly selected case (i.e., patients who initiated remdesivir), and assign an ‘Index Day’ representing the day of stay of the first remdesivir dose; 2) for each index Day, identify potential controls, including all patients who had not yet initiated remdesivir as of the Index Day, were still eligible for remdesivir based on laboratory values (eGFR, ALT, AST), and had a propensity score within one quarter of one standard deviation of the logit of the case patient’s propensity score, 3) if no candidate controls existed, the case patient was removed from analysis. If only one candidate control existed, that patient was selected and the match was considered final. If more than one candidate control existed, the mahalanobis distance between the case patient and the candidate controls was calculated based on the propensity score and patient characteristics prior to the index date. Specific characteristics included in the mahalanobis

© 2021 Ohl ME et al. *JAMA Network Open*.

distance were month of admission, ICU stay, oxygen saturation, mechanical ventilation use, and pulse. The candidate control patient with the smallest distance to the case patient was selected as the final match. This control patient was then removed from the possible pool of candidate matches for subsequent patients. The matching process was repeated iteratively for all patients who initiated remdesivir. The final sample included 559 cases with current or prior dexamethasone use matched to 559 controls, and 613 cases with no current or prior dexamethasone use matched to 613 controls. Our primary analysis of outcomes combined both matched sets resulting in 1172 cases matched to 1172 controls.

Chart review to verify COVID-19 as reason for hospital admission among controls

We sought to verify that hospitalizations for controls were related to COVID-19, as opposed to hospitalization for some other reason with an incidentally positive SARS-CoV-2 test on screening at admission. To address this issue, we reviewed a sample of 100 randomly selected charts from the propensity-matched cohort (50 remdesivir recipients and 50 matched controls) to confirm active COVID-19 as the primary reason for admission. We used VHA's Compensation and Pension Record Interchange (CAPRI) application to review patient charts to determine the clinical documentation of a diagnosis of acute COVID-19 during admission, or documentation from admission notes indicating clinical impression of an incidentally positive screening test on admission with an alternate reason for admission. Chart review was conducted by an infectious disease physician (author TK on manuscript) using a structured chart extraction form and review of all provider, nursing, and pharmacy notes, as well as laboratory values. Chart review revealed that 50/50 (100%) of remdesivir recipients and 49/50 (98%) of controls had a clinical diagnosis of COVID-19 as the primary active problem during admission. One of the 50 (2%) charts reviewed for controls revealed that the patient was admitted for complications of malignancy and had a positive screening SARS-CoV-2 polymerase chain reaction test at admission, but that the providers caring for the patient found no clinical evidence of symptoms potentially related to COVID-19.

© 2021 Ohl ME et al. *JAMA Network Open*.

Marginal structural models with inverse probability of treatment weighting

An alternative approach used marginal structural models (MSM) with stabilized inverse probability of treatment weights to estimate the relative impact of remdesivir on mortality and time to discharge.[4] MSM reduce bias by weighting the contribution of each patient during a given day by “stabilized” weights, where stabilized weights reflect both baseline and time-varying patient covariates. As with our primary analysis, the MSM approach used a dataset consisting of one record per patient per day to estimate probability of remdesivir initiation for each day of stay, until discharge or first remdesivir use. Using the approach of Hernan et al,[2] we estimated two separate propensity models: the first predicted first remdesivir use based on baseline patient characteristics only, while the second incorporated baseline as well as time-varying characteristics. The ratio of the two propensity scores are used to estimate stabilized inverse probability of treatment weights (IPTW) that vary by day of stay and effectively create a pseudo-population in whom patient covariates are no longer related to remdesivir initiation on a given day. Finally, we used generalized estimating equations with robust standard errors to fit the final weighted pooled logistic models to conduct weighed discrete time survival analyses, with day as unit of time, to estimate associations between remdesivir initiation and 1) 30-day mortality or 2) hospital discharge.

© 2021 Ohl ME et al. *JAMA Network Open*.

eFigure 1: Propensity matching scheme

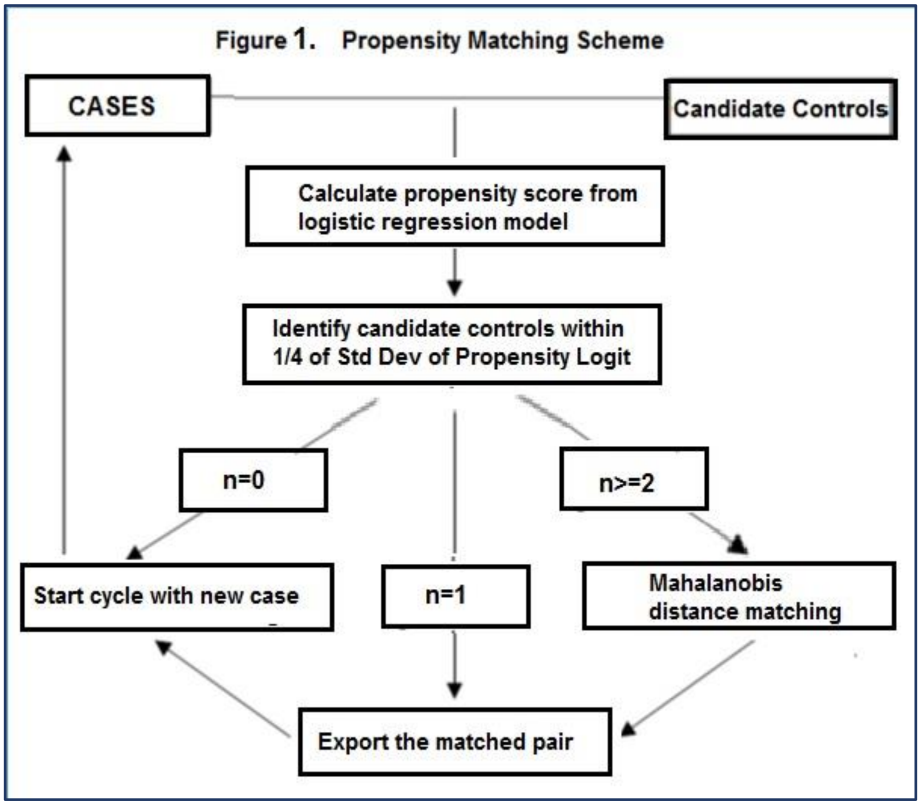

© 2021 Ohl ME et al. *JAMA Network Open*.

eFigure 2: Distribution of propensity scores among unmatched (0) and matched (1) remdesivir recipients

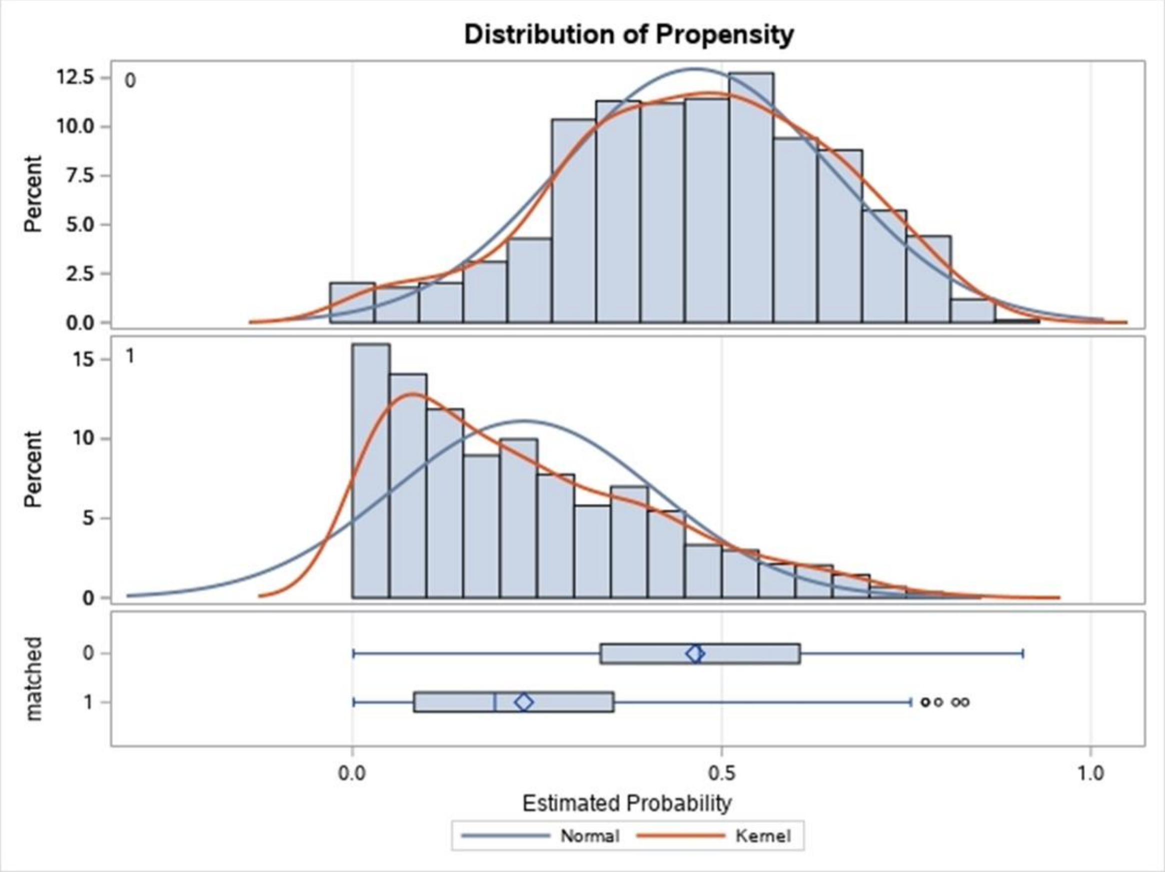

© 2021 Ohl ME et al. *JAMA Network Open*.

eTable2: Characteristics of matched and unmatched remdesivir recipients

among those with complete data on day of remdesivir initiation (N=2,011). All values are % on day of remdesivir initiation unless otherwise noted.

	Unmatched remdesivir recipients N=839	Matched remdesivir recipients N=1,172	p
Age, years, mean	68.8	66.6	<0.01
<55	11.3	19.5	<0.01
55-65	19.4	20.6	
65-75	41.5	33.0	
75-85	20.4	17.2	
> 85	7.4	9.7	
Female sex	5.7	6.1	0.69
Race/ethnicity			0.71
White	60.3	59.1	
Black	29.6	33.1	
Other	3.8	2.3	
Missing	6.3	5.5	
Admission month			<0.01
May	2.6	10.6	
June	9.2	19.6	
July	38.5	36.5	
August	28.3	16.9	
Sept-Oct	21.4	16.4	
PCR + on or before admit	99.8	99.0	0.10
Hospital day at initiation			
Day1	24.3	26.4	0.02
Day 2	44.6	33.5	
Day 3	15.4	17.7	
Day 4-5	10.1	13.4	
Day 6-8	4.3	6.2	
Day 9	2.1	2.9	
Comorbidity			
Myocardial infarction	11.1	9.0	0.11
Congestive heart failure	21.7	21.9	0.90
Peripheral vascular disease	16.2	18.3	0.23
Cerebrovascular disease	15.6	14.2	0.36
Arrhythmia	45.2	44.5	0.78
Hypertension	86.3	81.8	0.01
Diabetes	57.2	53.5	0.09
Chronic pulmonary disease	42.7	34.6	<0.01
Renal disease	28.7	25.4	0.09
Malignancy	18.8	13.4	<0.01
Liver disease	15.0	16.6	0.33
Dementia	12.3	14.5	0.15
Obesity	46.7	44.2	0.26
Alcohol diagnosis	9.4	13.1	0.01
Drug use diagnosis	6.6	9.9	0.01
ICU at initiation	33.8	20.7	<0.01

© 2021 Ohl ME et al. *JAMA Network Open*.

Mechanical ventilation at initiation	9.8	5.9	0.01
Percent O_2 saturation at initiation, mean	89.2	91.4	<0.01
O_2 saturation < 94% at initiation	92.4	81.4	<0.01
O_2 saturation < 94% ever prior to initiation	95.4	89.5	<0.01
Temperature at initiation, ºF, mean	99.5	99.4	00.44
Systolic BP at initiation, mmHg, mean	117.9	117.9	0.98
Diastolic BP at initiation, mmHg, mean	66.5	66.9	0.37
Respiratory rate at initiation, mean	24.3	22.9	<0.01
WBC at initiation, 10^3 cells/mm^3, mean	7.6	7.3	0.14
eGFR at initiation, ml/min, mean	70.4	74.5	<0.01
AST at initiation, IU/L, mean	48.1	46.7	0.31
ALT at initiation IU/L, mean	38.1	39.6	0.24
Dexamethasone at initiation	88.0	47.0	<0.01
Any corticosteroid at initiation	89.4	51.8	<0.01
Azithromycin at initiation	29.0	23.9	<0.01
Other antibiotic at initiation	40.9	34.6	<0.01
Heparin at initiation	17.2	14.1	0.06
LMWH at initiation	67.6	61.5	<0.01
Warfarin/DOAC at initiation	7.5	10.2	0.04
Warfarin/DOAC at admit	9.7	9.7	0.96
Famotidine at initiation	10.4	10.4	0.98
Famotidine at admit	3.5	4.0	0.52
Statin at initiation	52.6	42.3	<0.01
Statin at admit	53.8	46.4	<0.01
ACE inhibitor at initiation	13.7	11.0	0.07
ACE inhibitor at admit	27.4	23.0	0.03
ARB at initiation	8.6	6.7	0.10
ARB at admit	14.8	11.6	0.04
Hydroxychloroquine/chloroquine at initiation	0.1	0.5	0.04
Hydroxychloroquine/chloroquine at admit	0.4	0.8	0.24
Death within 30 days	17.9	12.2	<0.01

WBC – white blood cell
eGFR – estimated glomerular filtration rate
AST – aspartate aminotransferase
ALT – alanine aminotransferase
PCR – polymerase chain reaction
ICU – intensive care unit
ACE – angiotensin converting enzyme
ARB – angiotensin receptor blocker

© 2021 Ohl ME et al. *JAMA Network Open*.

eFigure 3: Kaplan Meier survival curves for matched cohort with dexamethasone use at match, N=1,118

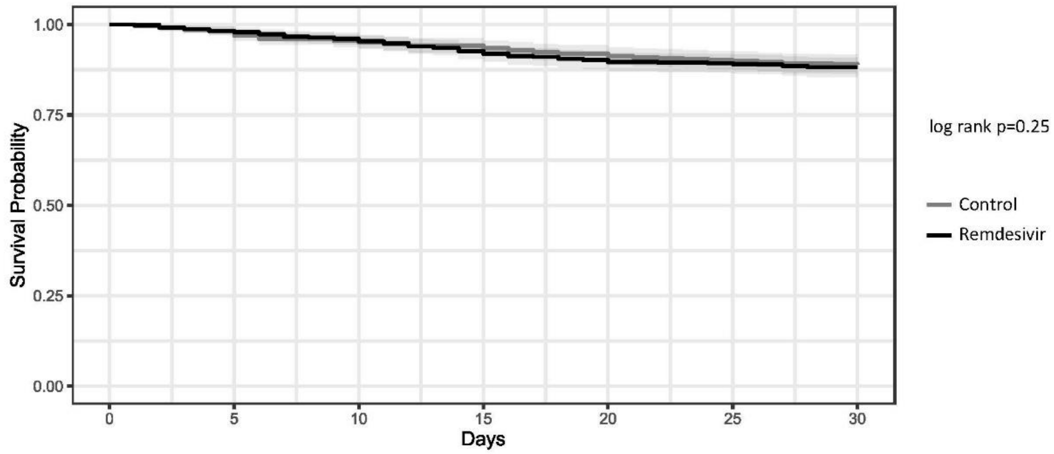

Control	559	534	519	509	497	490	484
Remdesivir	559	547	533	514	501	498	493

Number of Patients at Risk

© 2021 Ohl ME et al. *JAMA Network Open*.

eFigure 4: Kaplan Meier survival curves for matched cohort without dexamethasone use at match, N=1,226

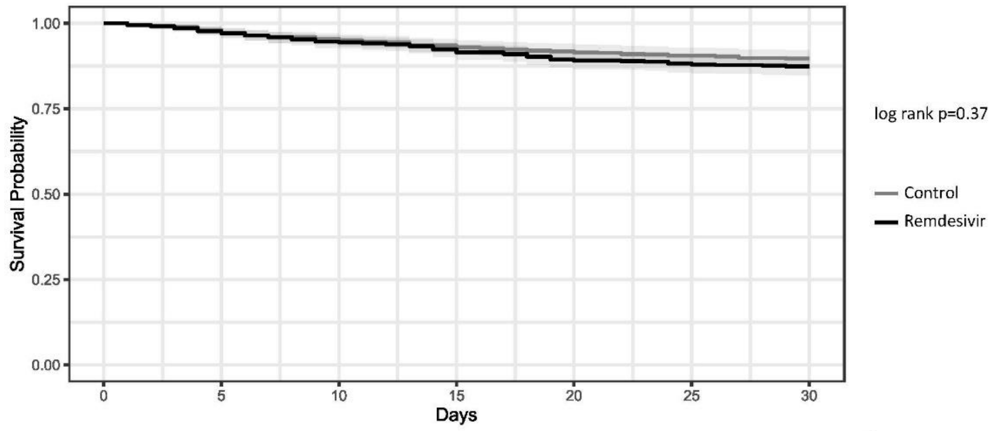

Control	613	595	579	566	557	551	546
Remdesivir	613	595	579	561	546	539	536

Number of Patients at Risk

© 2021 Ohl ME et al. *JAMA Network Open*.

eFigure 5: Cumulative incidence function plots for time to hospital discharge Day zero is day of match (i.e., day of remdesivir initiation or corresponding hospital day for controls)

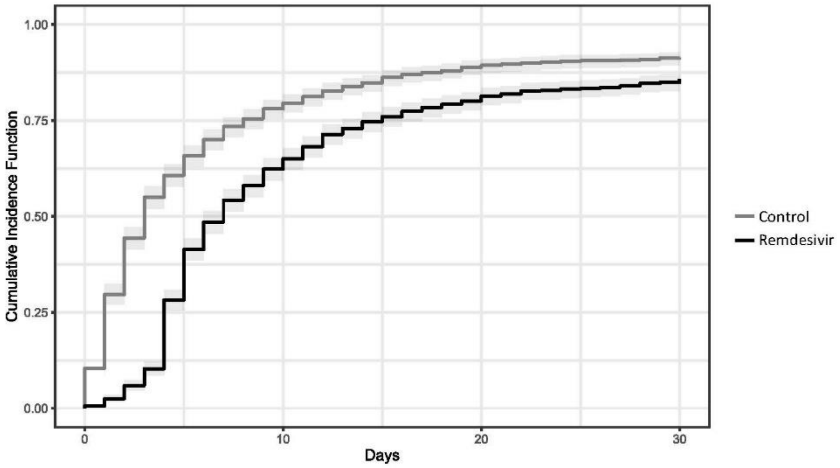

REFERENCES

1) Quan H, Sundararajan V, Halfon P, et al. Coding algorithms for defining comorbidities in ICD-9-CM and ICD-10 administrative data. *Med Care*. Nov 2005;43(11):1130-9
2) Veterans Health Administration. COVID-19: Shared Data Resource. Accessed June 3, 2020, https://vhacdwdwhweb100.vha.med.va.gov/phenotype/index.php/COVID-19:Shared_Data_Resource
3) Feng WW, Jun Y, Xu R. A method/macro based on propensity score and mahalanobis distance to reduce bias in treatment comparison in observational study. SAS Technical Papers, 2005.
4) Hernan MA, Brumback B, Robins JM. Marginal structural models to estimate the causal effect of zidovudine on the survival of HIV-positive men. *Epidemiology* 2000;11(5):561-70. doi: 10.1097/00001648-200009000-00012 [published Online First: 2000/08/24]

© 2021 Ohl ME et al. *JAMA Network Open*.
